# Kinetic mechanism of the dimeric ATP sulfurylase from plants

**DOI:** 10.1042/BSR20130073

**Published:** 2013-07-25

**Authors:** Geoffrey E. Ravilious, Jonathan Herrmann, Soon Goo Lee, Corey S. Westfall, Joseph M. Jez

**Affiliations:** Department of Biology, Washington University in St. Louis, One Brookings Drive, St. Louis, MO 63130, U.S.A.

**Keywords:** dead-end inhibition, enzyme, isothermal titration calorimetry (ITC), kinetic mechanism, plant sulfur metabolism, sulfur assimilation, APS, adenosine 5′-phosphosulfate, GmATPS1, *Glycine max* (soybean) ATP sulfurylase isoform 1, ITC, isothermal titration calorimetry, PAPS, adenosine 3′-phosphate 5′-phosphosulfate, PP_i_, pyrophosphate

## Abstract

In plants, sulfur must be obtained from the environment and assimilated into usable forms for metabolism. ATP sulfurylase catalyses the thermodynamically unfavourable formation of a mixed phosphosulfate anhydride in APS (adenosine 5′-phosphosulfate) from ATP and sulfate as the first committed step of sulfur assimilation in plants. In contrast to the multi-functional, allosterically regulated ATP sulfurylases from bacteria, fungi and mammals, the plant enzyme functions as a mono-functional, non-allosteric homodimer. Owing to these differences, here we examine the kinetic mechanism of soybean ATP sulfurylase [GmATPS1 (*Glycine max* (soybean) ATP sulfurylase isoform 1)]. For the forward reaction (APS synthesis), initial velocity methods indicate a single-displacement mechanism. Dead-end inhibition studies with chlorate showed competitive inhibition versus sulfate and non-competitive inhibition versus APS. Initial velocity studies of the reverse reaction (ATP synthesis) demonstrate a sequential mechanism with global fitting analysis suggesting an ordered binding of substrates. ITC (isothermal titration calorimetry) showed tight binding of APS to GmATPS1. In contrast, binding of PP_i_ (pyrophosphate) to GmATPS1 was not detected, although titration of the E•APS complex with PP_i_ in the absence of magnesium displayed ternary complex formation. These results suggest a kinetic mechanism in which ATP and APS are the first substrates bound in the forward and reverse reactions, respectively.

## INTRODUCTION

The most abundant environmental source of sulfur is sulfate (SO_4_^2−^), which is a chemically inert molecule [1,2]. For plants and microbes to utilize this essential nutrient, sulfate is enzymatically converted into a chemical species that is energetically favourable for reduction [3,4]. The sulfur assimilation pathway provides sulfide for a range of biosynthetic pathways that supply methionine, glutathione, iron–sulfur clusters, vitamin cofactors such as biotin and thiamin, and multiple specialized metabolites such as glucosinolates [1,2,5,6].

**Figure 1:**
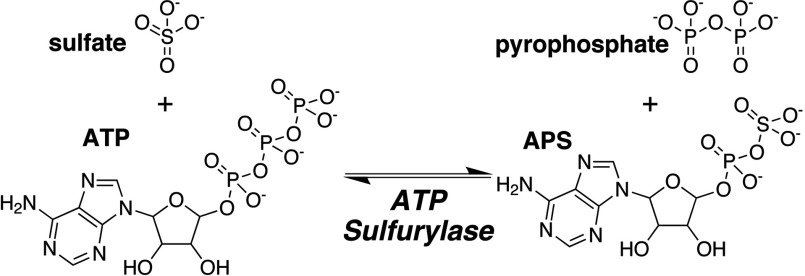
Overall reaction catalysed by ATP sulfurylase

The first enzymatic reaction in the sulfur assimilation pathway of plants is the non-reductive adenylation of sulfate catalysed by ATP sulfurylase (ATP: sulfate adenylyl transferase; EC 2.7.7.4) to yield APS (adenosine 5′-phosphosulfate) and PP_i_ (pyrophosphate) (Figure 1) [7]. Generation of the mixed phosphosulfate anhydride bond in APS yields a high-energy molecule that drives subsequent reduction reactions in the assimilatory pathway [8–11]. APS formation is energetically unfavourable (*K*_eq_~10^−7^–10^−9^) compared with the reverse reaction and requires high substrate concentrations and downstream enzymes to maintain the forward reaction equilibrium in the pathway [7,8]. Downstream enzymes in the plant sulfur assimilation pathway (i.e. APS reductase and sulfite reductase) convert APS into sulfite and then sulfide for subsequent incorporation into cysteine [1–4,6,12]. Alternatively, phosphorylation of APS to PAPS (adenosine 3′-phosphate 5′-phosphosulfate) supplies a sulfur-donor molecule for a variety of biosynthetic pathways [13–15]. In addition, coupling of the ATP sulfurylase reaction with hydrolysis of PP_i_ by pyrophosphatase helps to maintain APS synthesis *in vivo* [16].

In plants, gene families encode multiple isoforms of ATP sulfurylase with varied expression patterns and organelle localization [4,10,17–23]. The different genes encode plastidic and cytosolic forms of the enzyme in *Arabidopsis thaliana* (thale cress), *Brassica juncea* (Indian mustard), *Solanum tuberosum* (potato) and *Glycine max* (soybean) and share >60% amino acid sequence identity [4,17,18,20,22]. For example, the soybean genome contains four ATP sulfurylase genes (*Glyma10g3876*0, *Glyma20g28980*, *Glyma13g06940* and *Glyma19g05020*) encoding proteins with predicted plastidial and/or mitochondrial localization [4].

Biochemical analysis of a plastidic ATP sulfurylase from soybean [GmATPS1 (*Glycine max* (soybean) ATP sulfurylase isoform 1)] showed that the enzyme functions as a homodimer [10]. The oligomeric structure of the plant ATP sulfurylase differs from that of the enzyme from bacteria, fungi and mammals and also lacks allosteric regulation. Prokaryotic forms of ATP sulfurylase are heterodimeric proteins in which a GTPase subunit allosterically activates the catalytic subunit [24]. In fungi, such as *Saccharomyces cerevisiae* and *Pencillium chrysogenum*, ATP sulfurylase functions as a homohexamer in which each monomer contains an N-terminal ATP sulfurylase domain and a regulatory C-terminal APS kinase domain [25,26]. The human enzyme functions as a homodimeric protein with a reversed ATP sulfurylase/APS kinase domain order compared with the yeast enzyme [27]. In fungi and mammals, the fusion of two activities into a single protein allows for direct synthesis of PAPS, which is used as a substrate for subsequent sulfonation reactions [28]. Interestingly, in certain diatom and microalgae species, ATP sulfurylase and pyrophosphatase are also reported to function as a fusion protein [16]. In addition, potential interaction between ATP sulfurylase and APR reductase as a protein complex in onion suggests additional organization of enzyme activities in the plant sulfur assimilation pathway [29].

Owing to the differences in oligomeric organization, domain architecture and biochemical regulation between the ATP sulfurylases from plants and other organisms, here we investigate the kinetic mechanism of a plant ATP sulfurylase (i.e. GmATPS1) to understand the reaction sequence catalysed by this critical enzyme of the sulfur assimilation pathway in plants.

## EXPERIMENTAL

### Protein expression and purification

The pET-28a-GmATPSΔ48 bacterial expression construct, which encodes GmATPS1 as an N-terminal hexahistidine-tagged protein lacking the plastid localization sequence, was previously described [10]. Transformed *Escherichia coli* BL21(DE3) were grown at 37°C in Terrific broth containing 50 μg ml^−1^ kanamycin until A_600nm_~0.6–0.9. Following induction with 1 mM isopropyl 1-thio-β-D-galactopyranoside, cell cultures were incubated overnight (20°C; 250 rev./min). Cell pellets were prepared by centrifugation (10000 ***g***; 15 min) and then resuspended in 50 mM Tris (pH 8.0), 500 mM NaCl, 20 mM imidazole, 10% (v/v) glycerol and 1% (v/v) Tween-20. Cells were ruptured by sonication and the lysate cleared by centrifugation (45000 ***g***; 30 min). The lysate was loaded onto an Ni^2+^-nitriloacetic acid (Qiagen) column. After extensive washing with lysate buffer minus Tween-20, the bound protein eluted using 250 mM imidazole in wash buffer. Incubation with thrombin during overnight dialysis at 4°C against wash buffer removed the His-tag. Dialysed protein was reloaded on a mixed benzamidine–Sepharose/Ni^2+^–NTA column. The flow-through was loaded onto a Superdex-200 26/60 HiLoad FPLC size-exclusion column equilibrated in 25 mM Hepes (pH 7.5), 200 mM KCl, and 5% glycerol. Fractions corresponding to the major protein peak were pooled and examined for purity by SDS–PAGE. Protein was concentrated (Amicon) to 5–10 mg ml^−1^ with protein concentration determined using a molar extinction coefficient (ϵ_280nm_=54890 M^−1^ cm^−1^) calculated with ProtParam (http://web.expasy.org/protparam). Protein was flash frozen in liquid nitrogen and stored at −80°C until used.

For assays of ATP sulfurylase activity in the forward (i.e. APS synthesis) direction, *A. thaliana* APS kinase was used. Details of the generation of the expression construct, *E. coli* expression, protein purification, and enzyme assay for AtAPSK (*A. thaliana* APSK) were previously described [10,30–32].

### Enzyme assays

Initial reaction velocities were determined by observing the rate of change in absorbance of pyridine nucleotide at 340 nm (ϵ=6270 M^−1^ cm^−1^) in 500 μl systems at 25°C using a Beckman DU800 UV/vis spectrophotometer. The forward APS synthesis reaction used an assay system consisting of 50 mM Tris (pH 8.0), 15 mM MgCl_2_, 100 mM NaCl, 0.4 mM phosphoenolpyruvate, 0.2 mM NADH, 0.05 units of APS kinase, 20 units of pyruvate kinase and 30 units of lactate dehydrogenase. The specific activity of APS kinase (1.5 μmol min^−1^ mg^−1^) was determined spectrophotometrically, as described elsewhere [10]. The reverse ATP synthesis reaction used an assay system of 50 mM Tris (pH 8.0), 5 mM MgCl_2_, 1 mM NADP^+^, 1 mM glucose, 2 units of hexokinase and 1 unit of glucose-6-phosphate dehydrogenase. All reactions were initiated by addition of enzyme and were corrected for non-enzymatic rates.

For analysis of the bi bi substrate kinetic mechanism of GmATPS1, initial velocity rates were measured under standard assay conditions with a matrix of substrate concentrations. In the forward direction, assays used varied Na_2_SO_4_ and ATP concentrations. For the reverse reaction, initial velocities were determined using varied APS and sodium PP_i_ concentrations. The resulting data were analysed by global curve fitting in SigmaPlot (Systat Software, Inc.) to model the kinetic data to rapid equilibrium rate equations describing ordered sequential, *v*=(*V*_max_ [A] [B])/(*K*_A_ *K*_B_+*K*_B_ [A]+[A] [B]), and random sequential, *v*=(*V*_max_ [A] [B])/(α *K*_A_ *K*_B_+*K*_B_ [A]+*K*_A_ [B]+[A] [B]), kinetic mechanisms, where *v* is the initial velocity, *V*_max_ is the maximum velocity, *K*_A_ and *K*_B_ are the *K*_M_ values for substrates A and B, respectively, and α is the interaction factor if the binding of one substrate changes the dissociation constant for the other [33].

For the inhibition studies, initial velocities were determined in reactions containing chlorate (0–0.3 mM) and varied concentrations of either ATP or Na_2_SO_4_ in the forward reaction. The global fitting analysis of inhibition experiments used SigmaPlot to fit all data to the equations for competitive, *v*=*V*_max_/(1+((*K*_m_/[S]) (1+[I]/*K*_i)_)) or uncompetitive, *v*=*V*_max_/(1+[I]/*K*_i_+*K*_m_/[S]) inhibition [34].

### Calorimetric measurements

ITC (isothermal titration calorimetry) experiments were performed using a VP-ITC calorimeter (Microcal, Inc.). GmATPS1 was dialysed at 4°C in 25 mM HEPES, pH 7.5, 200 mM KCl, 5% glycerol. Stock solutions of APS and PP_i_ were prepared in the same buffer. Protein and ligand solutions were degassed at room temperature (22°C) prior to use. For each titration, 20–30 injections of 10 μl of ligand were added into sample solutions containing protein (18.5–50 μM) in the presence or absence of ligands. GmATPS1 complexed with APS was formed by incubating protein and 0.1 mM APS 30 min (4°C) followed by equilibration at 25°C before titration. Data were analysed using a one-site (i.e. identical sites) binding model, as follows:

$$Q_{i}^{\mathrm{tot}}=V_{0}•M_{i}^{\mathrm{tot}}•\left(\left(nK_{1}x\right)\Delta H_{1}\right)/1+K_{1}x),$$

where *Q_i_*^tot^ is the total heat after the *i*th injection, *V*_0_ is the calorimetric cell volume, *M_i_*^tot^ is the concentration of protein in the cell after the *i*th injection, Δ*H* is the corresponding enthalpy change, *n* is the number of nucleotide-binding sites per monomer, and *K* is the equilibrium-binding constant. Fitting of data was performed with Origin software.

## RESULTS

### Forward APS synthesis reaction: initial velocity analysis

The kinetic mechanism of the physiological APS synthesis reaction of GmATPS1 was first examined using a bi substrate variation experiment. For these experiments, GmATPS1 was expressed in *E. coli* as an N-terminally hexa-histidine tagged protein and was purified by nickel-affinity and size-exclusion chromatographies [10]. Three possible kinetic models–ping-pong, ordered sequential and random sequential describe two substrate to two product or bi bi kinetic mechanisms [33].

**Figure 2:**
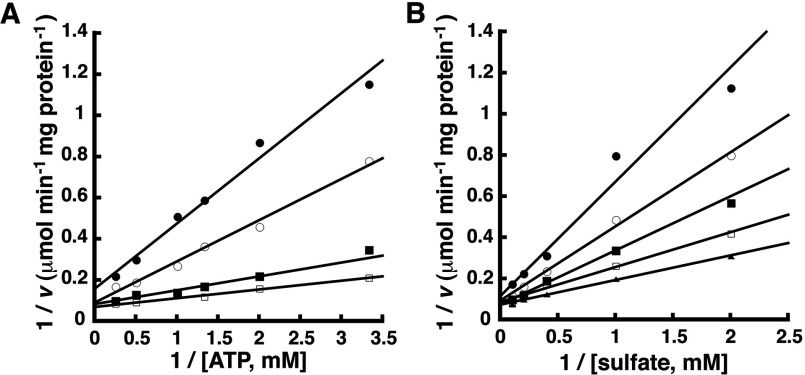
Initial velocity variation of substrates in the forward reaction Experimental data are shown as symbols in the double-reciprocal plots and lines indicate the global fit of all data to the equation for an ordered sequential bi bi mechanism. (**A**) Double-reciprocal plot of 1/*v* versus 1/[ATP] at 0.5, 1.0, 2.5 and 5 mM sulfate (top to bottom). (**B**) Double-reciprocal plot of 1/*v* versus 1/[sulfate] at 0.3, 0.5, 0.57 and 1.2 mM ATP (top to bottom).

Initial velocity data generated by co-varying the concentrations of ATP and Na_2_SO_4_ was used to distinguish between the three possible mechanisms (Figure 2). The primary pattern, in which the lines intersect, indicates that GmATPS1 uses a single displacement type of mechanism and eliminates the ping-pong type of mechanism from further consideration. The resulting data was globally fit to the equations describing the random and ordered bi bi substrate mechanisms. Examination of double-reciprocal plots, residual errors, standard errors of the fitted parameters, and the correlation coefficient evaluated quality of the fit to the observed data. Modelling of the initial velocity data to a random sequential mechanism yields a correlation coefficient (*r*^2^=0.989) slightly better than the fit to an ordered mechanism (*r*^2^=0.980), but the interaction factor (α) for the random mechanism was 9.53, which suggests that binding of one substrate is a negative interaction that decreases the affinity for the second substrate. Also, as discussed below, dead-end inhibition studies were consistent with an ordered mechanism. Thus, Table 1 summarizes the fitted kinetic parameters for an ordered sequential bi bi kinetic mechanism. The fitted kinetic parameters reported here were comparable with those previously published for GmATPS1 [10].

**Table 1:** Kinetic constants for an ordered sequential bi bi kinetic model

Reaction	Fitted parameters
Forward	
*V*_max_ (μmol min^−1^ mg of protein^−1^)	14.6±3.5
*K*_m_^ATP^ (μM)	221±41
*K*_m_^sulfate^ (μM)	1,030±280
Reverse	
*V*_max_ (μmol min^−1^ mg of protein^−1^)	58.8±11.5
*K*_m_^APS^ (μM)	28.3±0.8
*K*_m_^pyrophosphate^ (μM)	76.2±7.4

### Forward APS synthesis reaction: dead-end inhibition

To further examine the order of substrate addition in the forward reaction, chlorate (ClO_3_^−^) was used as a dead-end inhibitor. Chlorate has also been demonstrated in tissue culture to reduce sulfur assimilation pathway activity [35] and is a dead-end inhibitor of ATP sulfurylase from *P. chrysogenum* [36]. This approach was chosen instead of product inhibition studies because of the use of APS kinase in the coupling assay [8].

**Figure 3:**
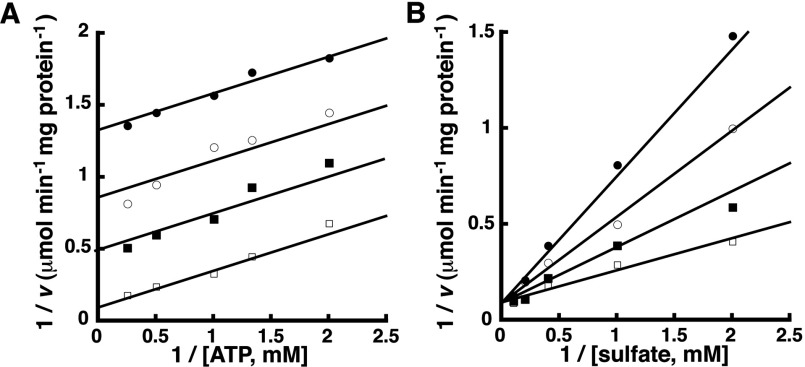
Chlorate inhibition in the forward reaction Experimental data are shown as symbols in the double-reciprocal plots and lines indicate the global fit of all data to the equation for either uncompetitive (**A**) or competitive (**B**) inhibition. (A) Double-reciprocal plot of 1/*v* versus 1/[ATP] at 0.3, 0.2, 0.1 and 0 mM chlorate (top to bottom). (B) Double-reciprocal plot of 1/*v* versus 1/[sulfate] at 0.3, 0.2, 0.1 and 0 mM chlorate (top to bottom).

The effect of oxyanions on the forward reaction catalysed by GmATPS1 was tested in the presence of chlorate, nitrate (NO_3_^−^) and carbonate (CO_3_^−^). Nitrate and carbonate were chosen because they are naturally present in the chloroplast and may regulate ATP sulfurylase activity [37]. In the presence of 100 μM chlorate, nitrate or carbonate, GmATPS1 displayed 35, 66 and 80% of wild-type APS synthesis activity, which is consistent with oxyanion inhibition reported for the fungal enzyme [36]. As chlorate was the most potent inhibitor of GmATPS1 it was used for inhibition studies of the forward reaction. Chlorate was an uncompetitive inhibitor (*K*_i_=132±4 μM) versus ATP (Figure 3A) and competitively inhibited (*K*_i_=103±19 μM) GmATPS1 with respect to sulfate (Figure 3B). The observed inhibition patterns indicate that ATP binds first, followed by sulfate in the forward reaction [38].

### Reverse ATP synthesis reaction: initial velocity analysis

**Figure 4:**
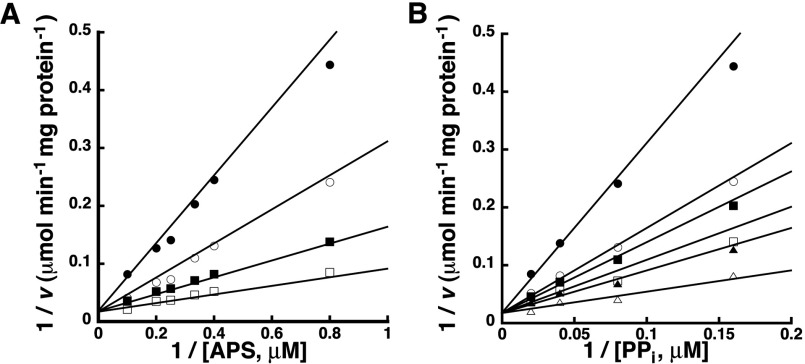
Initial velocity variation of substrates in the reverse reaction Experimental data are shown as symbols in the double-reciprocal plots and lines indicate the global fit of all data to the equation for an ordered sequential bi bi mechanism. (A) Double-reciprocal plot of 1/*v* versus 1/[APS] at 6.25, 12.5, 25 and 50 μM PP_i_ (top to bottom). (B) Double-reciprocal plot of 1/*v* versus 1/[PP_i_] at 1.25, 2.5, 3.0, 4.0, 5.0 and 10.0 μM APS (top to bottom).

Using the bi substrate variation approach described for the forward reaction, the ATP synthesis or reverse reaction catalysed by GmATPS1 was examined. Initial velocity patterns for the energetically favourable reverse reaction showed intersecting lines consistent with a sequential type of mechanism (Figure 4). Global fitting of the bi substrate data for the reverse reaction yielded a best fit to an ordered sequential mechanism (*r*^2^=0.961) compared with a random sequential mechanism (*r*^2^=0.909). The fitted kinetic parameters for the reverse reaction are summarized in Table 1 and are similar to previously reported values [10].

### Reverse ATP synthesis reaction: ITC analysis

To determine the order of binding, and thus the kinetic mechanism, in the reverse reaction, ITC was used. Product inhibition by ATP was complicated by the use of a coupling system and the binding of oxyanions (sulfate, chlorate, phosphate and phosphonoformate) was too weak to compete with the energetically favourable ATP synthesis reaction. Ligand-binding analysis provides a direct method for distinguishing between random versus sequential order of addition.

**Figure 5:**
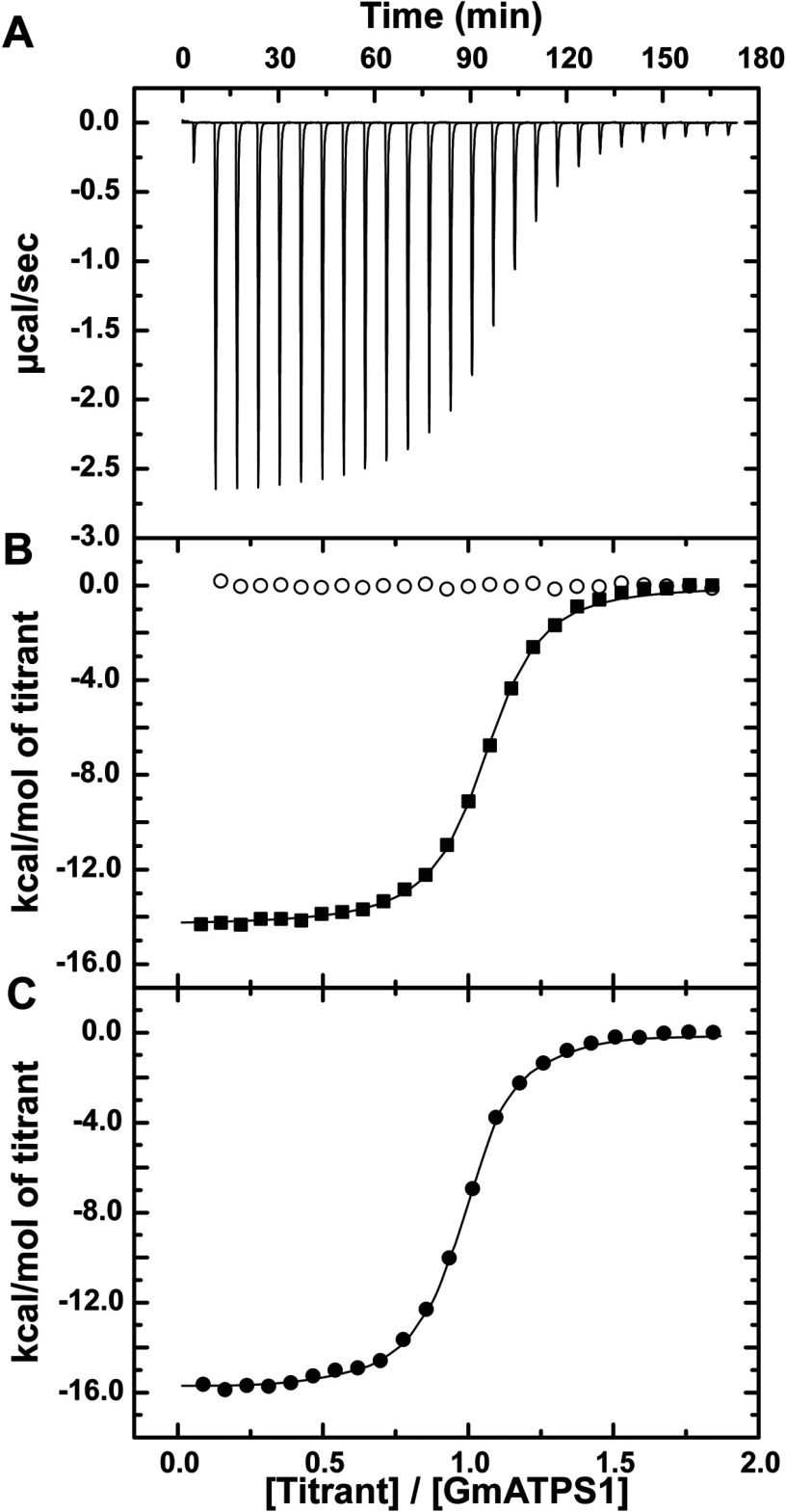
ITC analysis of ligand binding to GmATPS1 (**A**) Representative experimental data for the APS titration is plotted as heat signal (μcal s^−1^) versus time (min). Each experiment consisted of 20–30 injections of 10 μl each of ligand into a solution containing protein. (**B**) Titration of GmATPS1 with APS (solid squares) and PP_i_ (open circles). The integrated heat response is shown with the solid line representing the fit of the data to a one-site binding model. (**C**) Titration of the GmATPS1•APS complex with PP_i_. The integrated heat response is shown with the solid line representing the fit of the data to a one-site binding model.

Titrations of GmATPS1 with either APS or PP_i_ yielded clearly distinct results (Figures 5A,B). Addition of APS showed an exothermic heat signature indicative of tight binding to GmATPS1. Fitting of the data to a one-site binding model provides a determination of the *K*_d_ value that is comparable with the *K*_m_ of the reaction (Table 2). Titrations using PP_i_ as the ligand did not indicate interaction with GmATPS1, as no heat signature was detected (Figure 5B). These results were consistent with initial binding of APS, followed by PP_i_. To confirm this order of events, GmATPS1 was pre-incubated with APS, and then the E•APS complex was titrated with PP_i_. To prevent catalysis, Mg^2+^ was not added to either the ligands or protein [36]. As shown in Figure 5(C), this experiment resulted in a clear heat signature and allowed for the determination of thermodynamic parameters for formation of the ternary complex (Table 2). This suggests that formation of the E•APS complex is necessary for PP_i_ binding.

**Table 2:** Thermodynamic parameters of ligand binding to GmATPS1 All titrations were performed at 25°C as described in the Experimental procedures. ITC data were fit to a one-site binding model. ND=not detected.

Protein in cell	Titrant	*N*	*K*_d_ (μM)	ΔG (kcal mol^−1^)	Δ*H* (kcal mol^−1^)	-*T*Δ*S* (kcal mol^−1^)
GmATPS1	APS	1.03±0.01	0.44±0.02	-8.66±0.42	-14.4±0.1	5.69
GmATPS1	PP_i_	ND	ND	ND	ND	ND
GmATPS1•APS	PP_i_	0.96±0.01	0.30±0.02	-8.89±0.51	-15.8±0.1	6.92

## DISCUSSION

Sulfur, like nitrogen, phosphorus and potassium, is a macronutrient essential for plant growth, crop yields and resistance to pathogens [1–5,39]. In plants, ATP sulfurylase catalyses the thermodynamically unfavourable formation of a mixed phosphosulfate anhydride in APS as the first committed step in assimilation of environmental sulfur [8–11]. As part of this chloroplast-localized pathway, the activity of ATP sulfurylase needs to be coordinated with downstream enzymes to control flux through the system for maintaining sulfur supplies and to ensure that its products (APS and PP_i_) do not accumulate [10]. In contrast with the multi-functional, allosterically regulated ATP sulfurylases from mammals, fungi and bacteria, the plant enzyme functions as a dimeric protein and has not been as well studied biochemically [10,24–28]. Since engineering of plant sulfur assimilation has a number of potential biotechnology applications, understanding the biochemical control and chemistry of the enzymes in this pathway is useful for manipulating the pathway [4–6,39–42]. To better understand the molecular function of the plant ATP sulfurylase, we used a combination of initial velocity, inhibition and ITC-binding experiments to examine the kinetic mechanism of this enzyme.

Physiologically, ATP sulfurylase catalyses the formation of APS and PP_i_ from ATP and sulfate (Figure 1). Kinetic analysis of the forward reaction of GmATPS1 indicates that a single-displacement or ternary complex mechanism occurs (Figure 2). The intersecting families of lines for each substrate eliminates consideration of a ping-pong type of reaction, but simultaneous global data fitting did not adequately distinguish between the sequential versus random order kinetic mechanisms. For the inhibition studies of the forward reaction, the use of chlorate as a dead-end inhibitor was chosen over product inhibition analysis with APS and PP_i_ because of how tightly the products inhibit the enzyme and the effect of APS on the coupling enzymes in the assay [8,36]. The competitive inhibition versus sulfate and uncompetitive inhibition versus ATP observed for chlorate (Figure 3) is consistent with ATP binding first in an ordered mechanism. Based on the dead-end inhibition data, the kinetic parameters for an ordered sequential kinetic mechanism are reported in Table 1.

For the reverse or ATP synthesis reaction catalysed by GmATPS1, the bi substrate variation experiment agrees with the single-displacement mechanism observed in the forward reaction (Figure 4). In this case, global data fitting yielded a better agreement with an ordered sequential mechanism compared with a random ordered mechanism (Table 1). To distinguish between the two possible kinetic models, ITC was used to directly examine APS and PP_i_ binding to GmATPS1 (Figure 5). This approach could not be used in the forward reaction direction because of the lack of heat signature associated with binding of either ATP or sulfate to GmATPS1 (results not shown). Formation of GmATPS1 binary complex with APS showed tight-binding affinity and an exothermic interaction (Figure 5B and Table 2). In contrast, no heat signature was detected in titrations using PP_i_ (Figure 5B and Table 2), even though both substrates for the reverse reaction have comparable kinetic parameters (Table 1 and [10]). To confirm binding of PP_i_ to the E•APS complex, titration to the complex was performed in the absence of magnesium to prevent catalysis [36]. Unlike the PP_i_ titration to free enzyme, a strong heat signature was detected for formation of the binary complex (Figure 5C and Table 2). These results suggest an ordered sequential mechanism for the reverse reaction.

**Figure 6:**
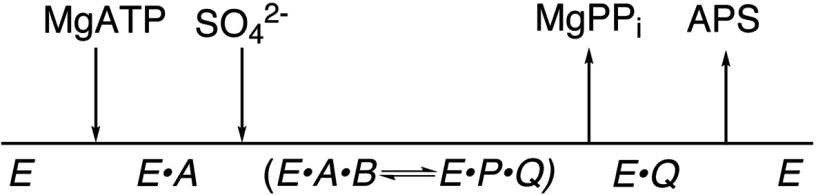
Model for the ordered sequential kinetic mechanism of plant ATP sulfurylase

An overall kinetic mechanism for GmATPS1, and the related plant enzymes, is now proposed (Figure 6). In the forward reaction, an ordered mechanism in which sulfate binding follows initial binding of ATP is most consistent with the initial velocity and dead-end inhibition data. For the reverse reaction, the initial velocity analysis and ITC-binding studies indicate that APS binding occurs first, followed by addition of PP_i_.

To date, kinetic studies of the ATP sulfurylases from mammals, fungi and plants have led to two different models for binding of substrates [8,36,43]. Initial velocity and inhibition studies of the enzyme from rat and *P. chrysogenum* were consistent with an ordered addition of substrates in each reaction direction [36,43]. In contrast, studies with the spinach enzyme suggest a random addition of substrates in the forward reaction and an ordered addition in the reverse reaction [8]. The kinetic analysis of the soybean enzyme presented here and earlier work with the spinach enzyme [8] differ in the proposed models for the forward reaction; however, analysis of the spinach enzyme used a molybdolysis reaction and not the physiologically relevant chemistry. Moreover, it is possible that the random mechanism exhibits a preferential binding order, which appears as an ordered mechanism using steady-state kinetic approaches. For example, with the plant APS kinase, steady-state kinetic studies suggested that the enzyme used an ordered mechanism for catalysis [44]. Later, extensive analysis of ligand binding by ITC demonstrated that each substrate interacts with the free enzyme to form binary complexes (i.e. random addition), but that the order of substrate addition dramatically alters affinity for the second ligand to form the ternary complex [30–32]. As noted above, global fitting of the initial velocity data for the forward reaction catalysed by GmATPS1 did not distinguish between the two possible sequential mechanisms and the interaction factor obtained for a random mechanism suggested that binding of one substrate decreases affinity for the second. Nevertheless, the dead-end inhibition pattern observed with GmATPS1 using chlorate supports an ordered sequential mechanism.
